# Using inverse finite element analysis to identify spinal tissue behaviour in situ

**DOI:** 10.1016/j.ymeth.2020.02.004

**Published:** 2021-01

**Authors:** Marlène Mengoni

**Affiliations:** Institute of Medical and Biological Engineering, School of Mechanical Engineering, University of Leeds, Leeds LS2 9JT, UK

**Keywords:** Optimisation, In silico models, Data variability

## Abstract

In computational modelling of musculoskeletal applications, one of the critical aspects is ensuring that a model can capture intrinsic population variability and not only representative of a “mean” individual. Developing and calibrating models with this aspect in mind is key for the credibility of a modelling methodology. This often requires calibration of complex models with respect to 3D experiments and measurements on a range of specimens or patients. Most Finite Element (FE) software’s do not have such a capacity embedded in their core tools.

This paper presents a versatile interface between Finite Element (FE) software and optimisation tools, enabling calibration of a group of FE models on a range of experimental data. It is provided as a Python toolbox which has been fully tested and verified on Windows platforms. The toolbox is tested in three case studies involving *in vitro* testing of spinal tissues.

## Highlights

• Versatile interface between optimisation tools and Finite Element solver developed.
• Capacity demonstrated for a range of objective functions.
• Applications detailed for the identification of material parameters in the spine.
• Toolbox shared under GPL license at https://github.com/mengomarlene/opti4Abq.

## 1 Introduction

In the area of pre-clinical testing of medical devices, *in silico* modelling is a promising tool, with the ability to model very specific situations, to account for disease-specific tissue behaviour, and more generally to provide a testing platform that can account for the large variation in the population [1]. Key requirements for *in silico* models to become mainstream are the assessment of their credibility defined from a clear understanding of their applicability [2] and known calibration and validation experiments [3], [4].

Modelling bone and fibrocartilage is of particular interest to improve the understanding of a range of musculoskeletal diseases, their progression for a particular individual, and the potential for success of surgical interventions. In a purely mechanical point of view, natural tissue behaviour is often modelled with a phenomenological approach [5], [6]. A phenomenological model has the benefit of being relatively simple with respect to the description of complex tissues, with the disadvantage that parameters are not directly measurable and need to be calibrated to match experimental data. However, because natural tissues are anisotropic, hydrated, and with in situ pre-strains [6], [7], [8], [9], conducting experimental analysis with standard mechanical tests in order to directly derive a stress/strain behaviour is often not representative of the physiological behaviour of the tissue. The calibration of constitutive models directly from experimental data is therefore not always possible or relevant.

Combining *in vitro* or *in vivo* testing with *in silico* modelling in such a way that the experimental testing is exactly replicated in specimen- or patient-specific *in silico* models has the advantage to provide direct comparisons, which can be used to calibrate *in silico* models while representing the variability between specimens or patients [3], [8]. In purely mechanical models, the Finite Element (FE) Method of Analysis is often a preferred method when the outcomes of interest are related to tissue strains or to the distribution of contact pressure. Developing inverse analysis tools is crucial for the quick and efficient calibration of FE models in complex 3D scenario.

Most FE software, commercial or open-source, provide the ability to calibrate one model to one set of experimental data, sometimes restricting the type of data which can be directly compared. However, given the natural variability in experimental testing outcomes, it can be beneficial to run simultaneous calibration across a range of specimens or experimental measures, expanding beyond in-built capacities; which requires new tools. The calibration in itself is an optimisation process, aiming to minimise the difference between experimental data and *in silico* data. It requires the interaction between FE software’s inputs and outputs and optimisation algorithms.

This paper presents and tests a versatile interface between FE software and optimisation tools, enabling calibration of a group of FE models on a range of experimental data.

## 2 Materials and methods

For historical reasons, the FE software used in this work was Abaqus (Simulia, Dassault Système), which possesses a scripting interface for Python 2.7. Optimisation algorithms chosen were thus those available in the scipy.optimize Python package (scipy 0.18 for Python 2.X). The tools described here were only fully-tested on Windows platforms.

### 2.1 Toolbox description

The opti4Abq toolbox [10] was developed to provide a bridge between an FE solver and an optimisation toolbox, using optimisation algorithms for univariate or multivariate functions or functionals, with parameter values bounded or not. It consists of only two classes: one managing the optimisation method, the other managing the objective function. The user needs only to create one object and call one function from the class the object is instantiated from (see example files shared with the toolbox). This object takes for attributes the path of the FE input files, the path of corresponding experimental data to optimise for, initial values of parameters to optimise (except for univariate optimisation of scalar functions), and bounds for those parameters if required (in this case enabling the use of a constrained optimisation method). Each FE input file is a Python file containing (1) a function which defines an FE model as a function of the parameters to optimise for, and (2) a function to post-process results into a value of interest in the form of a scalar, a list, or a curve (i.e. two lists of the same size as (x,y) data). When the optimisation is to be run for several models, each FE input file needs to be located within one directory; corresponding experimental data needs to be named with the file names used for the FE model files (with a .dat extension). In the particular case where the FE solver is Abaqus, using the in-built Python scripting interface eases the definition of the FE model and of the processing of the output database.

All optimisation algorithm interfaced in the optimisation class are gradient-based minimisation methods. Depending on the nature of the data to optimise for, the number of parameters, and whether they are bounded or not, the toolbox calls a specific optimisation algorithm (Table 1). Options controlling the maximum number of iterations, the termination criteria for the objective function (normal termination), the parameter variation, and the gradient, as well as the step size used to compute the numerical gradient of the objective function, can be used if necessary.

**Table 1:** Optimisation methods used in opti4Abq toolbox (more information on the methods can be found in the online documentation of scipy.optimize [11]).

**Multivariate optimisation**		

Parameters	**Scalar** Objective Function	**Functional** Objective Function

**Constrained**	Limited-memory Broyden-Fletcher-Goldfarb-Shanno Bounded (L-BFGS-B) Method	Trust Region Reflective Method

**Unconstrained**	Conjugate Gradient Method	Levenberg-Marquadt Method

**Univariate optimisation of a scalar objective function** (constrained or not)		

Brent Method		

Within each optimisation algorithms, FE models are run and post-processed as defined in the FE input files, returning outcome values which are compared (either directly or in the least square sense, as a difference or a relative error) with the corresponding experimental data. When the outcome of interest is (x,y) data and when the FE models do not return y-values for the same x-values as the experimental data, the data is first interpolated to the largest of the experimental or computational sampling rate so that y-values can be directly compared as a list. When the FE solver does not converge, it can return “NaN” which is handled by the optimisation if a series of different models are used within the same optimisation process and at least one model has converged.

When terminated, the optimisation returns the final values of the parameters, the value of the objective function, the number of iterations and function evaluations that were required, and information about the criterion reached for termination of the optimisation process. It can also return the value of the estimated Jacobian and Hessian matrices. The user can also choose to return in text files the history of the parameters and objective function values for each iteration of the process. FE models and output databases are overwritten at each iteration, making the process not costly in terms of data space required.

### 2.2 Case studies: Material parameters identification in the spine

**Fig. 1:**
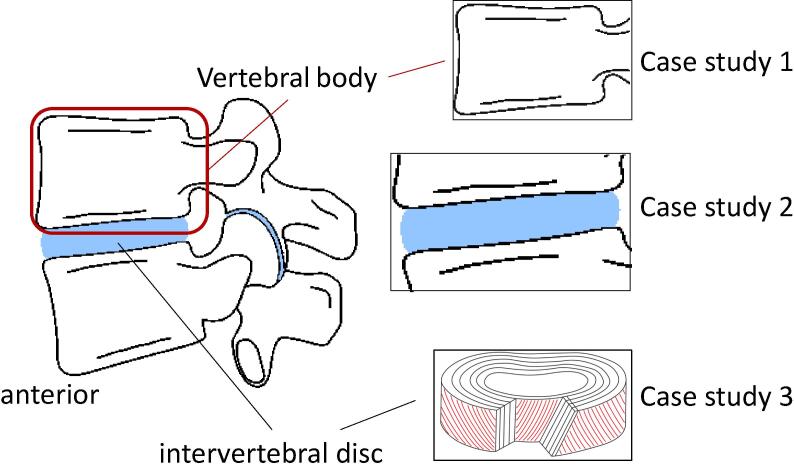
The functional spinal unit comprises of two vertebrae and one intervertebral disc; three case studies are presented (1) vertebral body only; (2) a bone-disc-bone segment; (3) a radial slice of the intervertebral dis.

The functional spinal unit (Fig. 1) is a good example to highlight the versatility of the tools developed, using a range of optimisation algorithms for a range of tissues and structures of interest. In all cases, parameters of interest are material parameters of constitutive models, which all need to be positive: only constrained optimisation methods were used.

All raw data is available from the original case studies [12], [13], [14]; processed data is available from the University of Leeds data repository [15]. Each case study is available as documented example files distributed with the toolbox [10].

#### 2.2.1 Bone elasticity function of CT image greyscale

In modelling bone, it is often of interest to capture the spatial variation in bone density; this is usually done by mapping material properties to 3D image data (such as CT). When the interest in modelling bone is only in its apparent linear stiffness, it has been shown that a linear mapping between CT greyscale and material elasticity is valid [16], if the relationship is calibrated for the experimental and imaging conditions and site of interest.

Calibration of FE models was run for 22 ovine vertebral bodies, all experimentally tested in similar conditions, with relatively high variance in the experimental data (coefficient of variance of 47%). FE models were built from microCT scans at 82-µm isotropic resolution, replicating experimental loading conditions. Output of choice for the model was the ratio of applied force to displacement, defining an apparent stiffness. Research data and discussion from this example is available from previously published work [12], [17].

Data for comparison between experiments and models was the stiffness (a scalar) and only the linear mapping needed to be calibrated (one scalar parameter). Hence, the Brent method was used (univariate scalar function, Table 1), with the RMS normalised difference across all samples used as objective function. For comparison purposes, a standard Newton method was also used to solve the optimisation process. Normal termination of the optimisation process was achieved when the objective function reached a value below 0.1.

The ability to calibrate a simple linear relationship across a range of different samples from normalised CT greyscale values allows us to use the process in a standardised manner, providing a framework to develop such models regardless of scanner settings and equipment used. It has allowed demonstration of large variability between species in the mapping of material properties to 3D image data [17].

#### 2.2.2 Derivation of material parameters in functional intervertebral disc models

The intervertebral disc is composed of different tissues which are all fully integrated between each other and to the bone. In computational models aimed at replicating the mechanical function of the intervertebral disc in compression experiments, it has been shown [8] that using a structural constitutive model of the disc is sufficient if the fibre-reinforced part of the disc model is calibrated against whole disc experimental data specific to the species tested. In the present case, this is two material parameters in a GOH hyperelastic constitutive model [18] which describe an exponential behaviour of fibres in tension only. Other components of the constitutive model (fibre orientation and dispersion, ground matrix shear modulus, and compressibility) were assumed to be known.

Calibration of FE models was run for six bovine osteodiscs (one intervertebral disc surrounded by two half vertebrae), all experimentally tested in similar conditions, with relatively low variance in the experimental data. FE models were built from microCT scans at 74-µm isotropic resolution, replicating experimental loading conditions. Output of choice for the model was the whole non-linear force/displacement behaviour. Research data and discussion from this example is available from previously published work [8], [13].

Data for comparison between the experiments and model was the RMS difference between loads vs displacement curves (a scalar), and two material parameters needed to be calibrated. Hence, an L-BFGS-B method was used (multivariate scalar function, Table 1), with the RMS difference across all six samples used as objective function (group calibration). For comparison purposes, calibration was also run on each sample individually. Normal termination of the optimisation process was achieved when the objective function reached a value below 5% of the largest applied load.

The ability to reverse-engineer material properties for one or several specimens has provided the capacity to evaluate the intra-specimen variation of parameters which are difficult to measure experimentally, or which do not have a direct physical interpretation. The ability to capture this variation increases confidence that the validation process captures some of the intrinsic variation in the specimens rather than being able to represent an average specimen only.

#### 2.2.3 Identification of requirements to model interface behaviour

The outer tissue of the intervertebral disc is composed of several lamellae of highly oriented collagen fibres embedded into an extrafibrillar matrix. Each lamella is bound to the next through an interlamellar network of elastin. Analysing details of damage processes of the intervertebral disc requires an understanding of the mechanical behaviour of this interlamellar network [19], which cannot be excised from the tissue for direct testing. Its behaviour can however be derived from experiments testing the disc in directions for which the collagen fibres bear minimum load.

Calibration of FE models was run for three radial slices of ovine intervertebral disc, obtained through microtome sectioning and tested in tension under light microscopy (60 µm thickness). FE models were built in 2D from the microscopy images and displacement of the interlamellar junctions was recorded. Research data and discussion from this example is available from previously published work [14], [20].

Data for the comparison between the experiments and model was the radial displacement of between eight to sixteen predefined points (a list), and the radial and tangential stiffness values of the lamellar interfaces were calibrated (two parameters). Hence, a trust region reflective method was used (multivariate functional, Table 1), with the normalised difference in displacements in each direction for each point used as objective function. Normal termination of the optimisation process was achieved when the objective function reached a value below 0.01. Optimisation was run independently for the three samples.

This ability to reverse-engineer interface properties in the case of intricate connection between tissues allows for the development of models that can study the degradation of such connections, in particular due to surgical interventions, for which local disruption of tissue interface may have large effects.

## 3 Results

For all three case studies, details of the results and discussion on their range of validity can be found in corresponding papers [8], [17], [20]. The results presented here are those specific to the optimisation process.

The optimisation process for all case studies terminated with parameter values within the range of expected physical values.

**Fig. 2:**
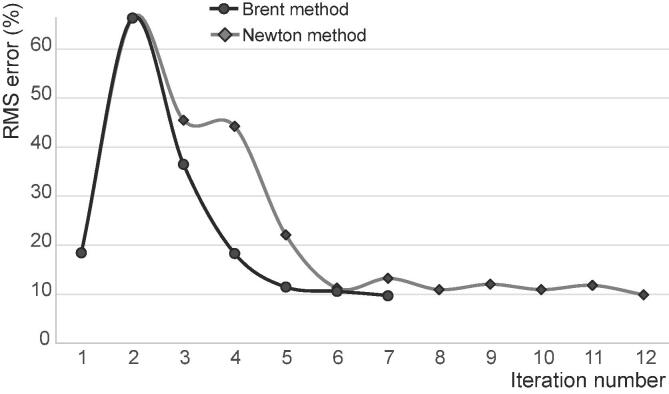
Convergence of the optimisation process: Brent vs Newton method.

**Fig. 3:**
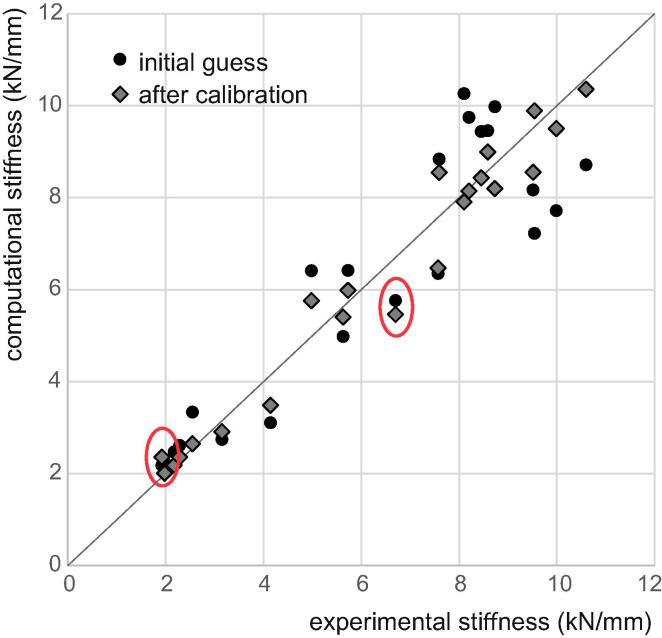
Outcome of calibration for the bone elasticity with the Brent method. The red ellipses highlight the two samples for which the group calibration did not improve the local difference.

For the bone elasticity case, seven iterations were necessary for completion using the Brent method and 12 using a Newton method (Fig. 2). For the Brent method, each iteration requires two or three function evaluations (with each function evaluation involving 22 FE models to run), for a total of 19 function evaluations. The Newton method requires two function evaluations per iteration, for a total of 24 function evaluations. The Brent optimisation led to improvements in the RMS difference (objective function) and in the one-to-one difference of all but two samples (Fig. 3), reducing the RMS error from 18.4% to 9.7% (termination was set at 10%) and maximal one-to-one error from 31% to 23%.

**Fig. 4:**
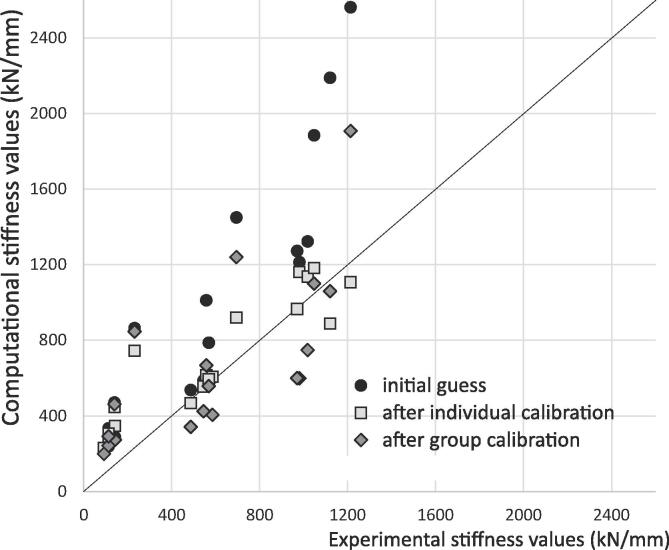
Outcome of calibration for the functional intervertebral discs with the L-BFGS-B method (figure adapted from [8]). The stiffness values are those of a tri-linear fit of the load/displacement data.

For the functional intervertebral disc models, the group optimisation converged for the parameter values, not the objective function, reducing the RMS difference in loads from 775 N to 320 N. It required six iterations of the L-BFGS-B method, for a total of 33 function evaluations (each evaluation requiring six FE models to run). The optimisation performed separately on each sample led to a convergence of the objective function for four models (normal termination) and of the parameter values for the two remaining models, requiring between 12 and 36 function evaluations per sample. In all cases however, it led to appropriate concordance correlation coefficients of the piecewise stiffness values between experimental and computational data, improved from 0.61 to 0.70 for the group optimisation and 0.86 for the individual optimisations (Fig. 4).

**Fig. 5:**
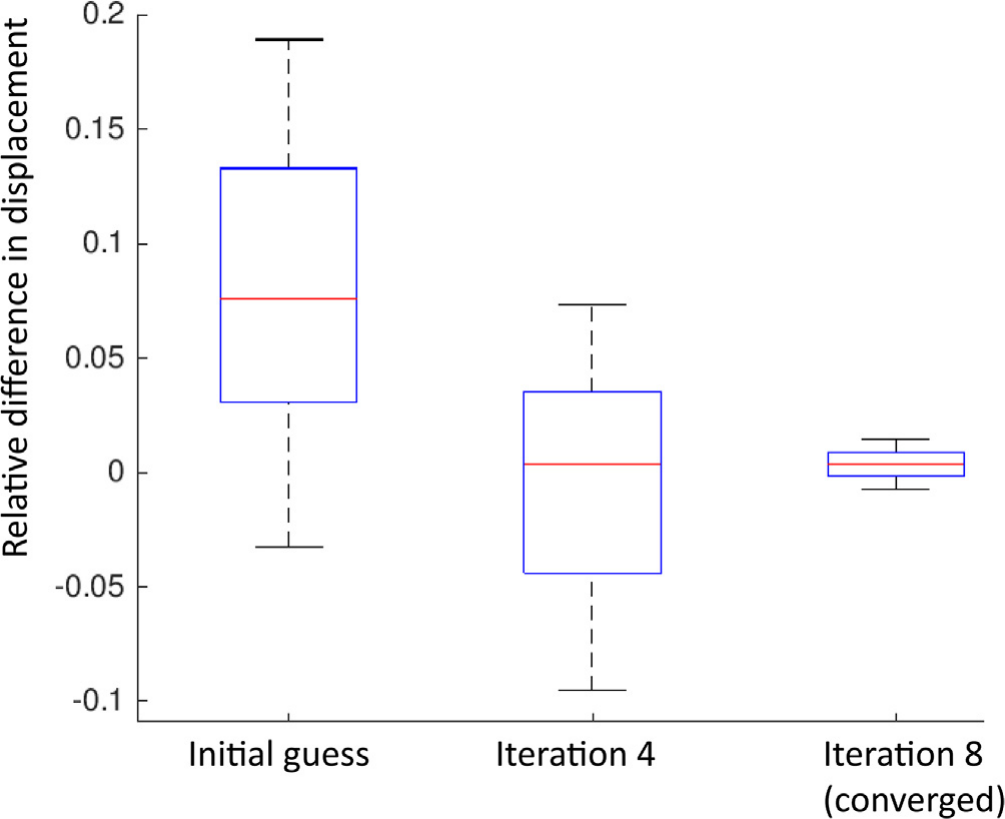
Outcome of calibration for radial slices of intervertebral discs with the trust region reflective method: each box plot represents the relative difference in local displacements across all points of interest for the three models.

For the interface intervertebral disc model (Fig. 5), 8 iterations per sample were necessary for normal completion of the trust region reflective method, requiring a total of 36 to 52 function evaluations each (with each function evaluation involving one FE model to run).

## 4 Discussion

The developed toolbox is composed of two classes defining objects respectively for the objective function and the optimisation process. Objects instantiated from the objective function class are independent from the optimisation tools used, and similarly those instantiated from the optimisation process class are independent of the FE solver used. Only two functions of the objective function class are specific to Abaqus and can be extended by setting up a solver attribute and replacing the command lines required to call this solver. For FE solvers which do not use Python as a scripting interface, the functions in the input file of each FE model would need to call the pre-processor and post-processor tools for the specific FE solver used.

The toolbox has been tested only on Windows platforms and has been extended to Unix platforms (using a switch for command-line based actions) with preliminary beta-testing. It is known to not be easily adaptable to time-restricted highly parallel platforms given the number of function evaluations cannot be estimated and each function evaluation can require long processing times (directly dependent on the complexity and number of FE models to run). The opti4Abq toolbox [10] is distributed on GitHub under GPL license, with documentation and example files which are reduced versions (in terms of number of models) of the three case studies presented in this paper.

Whereas all presented case studies used material parameters as parameters to calibrate, the toolbox can, in theory, be used for any type of continuous parameter in a model. The optimisation algorithms currently interfaced are sufficient for pragmatic modelling, where the number of parameters to optimise for is relatively low and initial values of these parameters can be well estimated. Other methods, including global optimisation methods, may need to be interfaced for less well-defined problems or for completely unknown parametric values for which a parametric sweep to define a range of values may not always be practical. Confidence intervals for the evaluated parameters can be estimated from the covariance matrix, computed by combining the Hessian matrix and the residuals.

By allowing to run simultaneous optimisation of a series of FE models and values of interest, the developed toolbox adds on existing 1-on-1 calibration capacity of most FE solvers (such as available in FEBio [21]). For Abaqus, the only in-built material calibration functionality works from stress/strain data and not inverse-FE models; the extended SIMULIA suite with Isight could be used to interface similar optimisation tools. Similar work can also be found in GIBBON [22], a MATLAB toolbox which interfaces with FEBio or Abaqus. The functions in GIBBON could be adapted to enable simultaneous optimisation of several models.

The tools presented in this paper were crucial in being able to calibrate image-based Finite Element Models representative of experimental tests. By calibrating for more than one specimen at a time, understanding of the intrinsic variation between specimen was gained, independently of their specific anatomy, which is not possible by analysing the experimental data directly. Such calibration, when followed by validation experiments on the same values of interest for different specimens, defines a clear range of applicability of the modelling methodology, which is the first step in defining a context of use for *in silico* models.
